# Supplementary material for: Emergent functions of noise-driven spontaneous activity: homeostatic maintenance of criticality and memory consolidation
Source: Front Neural Circuits. 2025 Oct 23;19:1585087. doi: 10.3389/fncir.2025.1585087 (PMC12589027; doi:10.3389/fncir.2025.1585087)
Supplement: Supplementary file 1 [file Data_Sheet_1.pdf]

Supplementary Information for

Emergent functions of noise-driven spontaneous activity: Homeostatic maintenance of criticality and memory consolidation

Narumitsu Ikeda, Dai Akita, Hirakazu Takahashi

Correspondence to: Hirokazu Takahashi

Corresponding author. Email: takahashi@i.u-tokyo.ac.jp

This PDF file includes:

Supplementary Figure S1 to S7

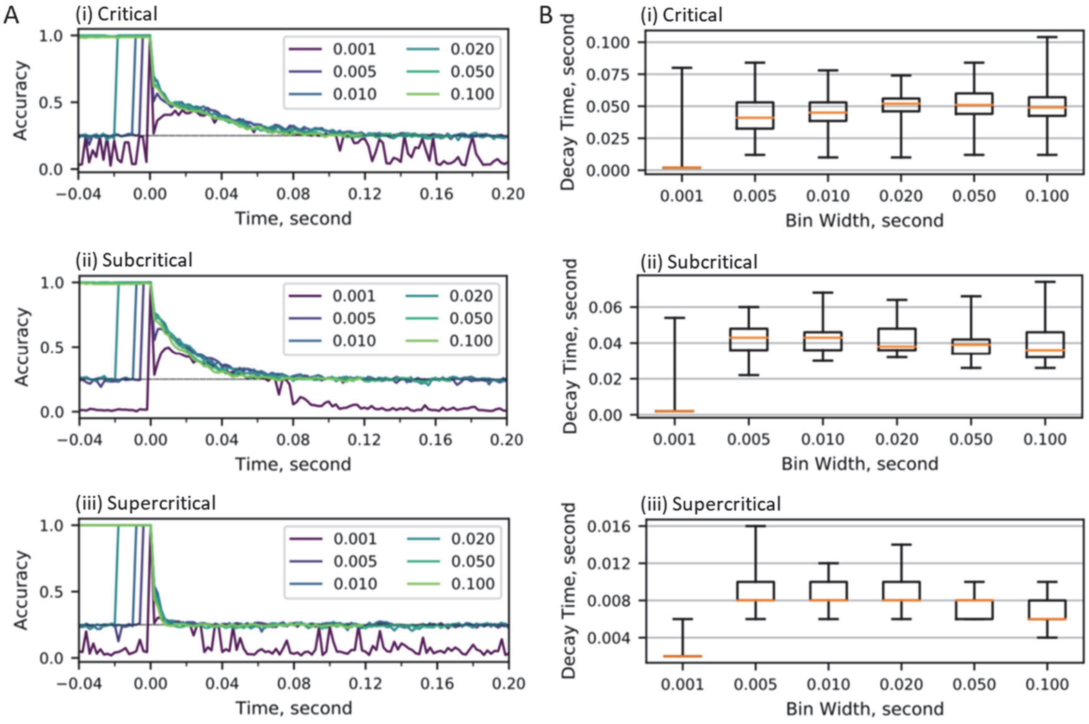

Supplementary Fig. S1 Effects of time-bin width on fading memory analysis. (A) Traces of decoding accuracy over time. (i) Critical SNN. (ii) Subcritical SNN. (iii) Supercritical SNN. (B) Decay times of the fading memory.

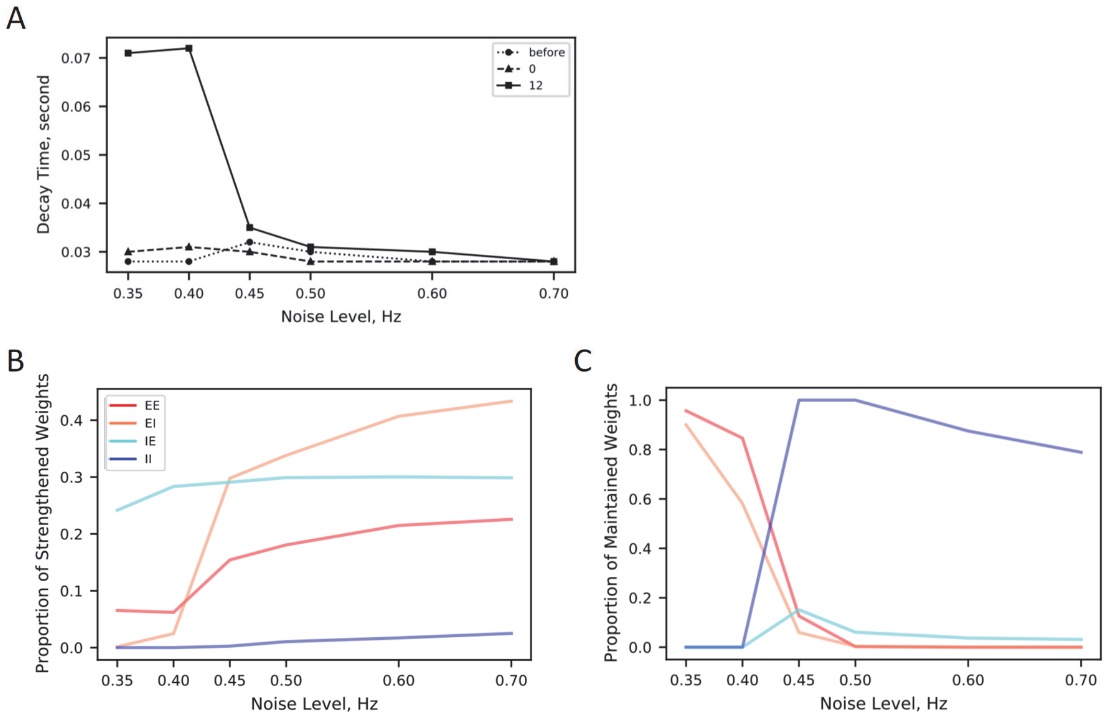

Supplementary Fig. S2 Effect of noise level on fading memory prolongation. (A) Decay times of SNNs as a function of the noise level. The medians of the decay times before, immediately after, and 12 h after repetitive stimulation are shown. Optimal fading memory prolongation was observed at a noise level of 0.40 Hz. (B) Proportions of weights strengthened by repetitive stimulation as a function of the noise level. (C) Proportions of weights maintained for 12 h after repetitive stimulation.

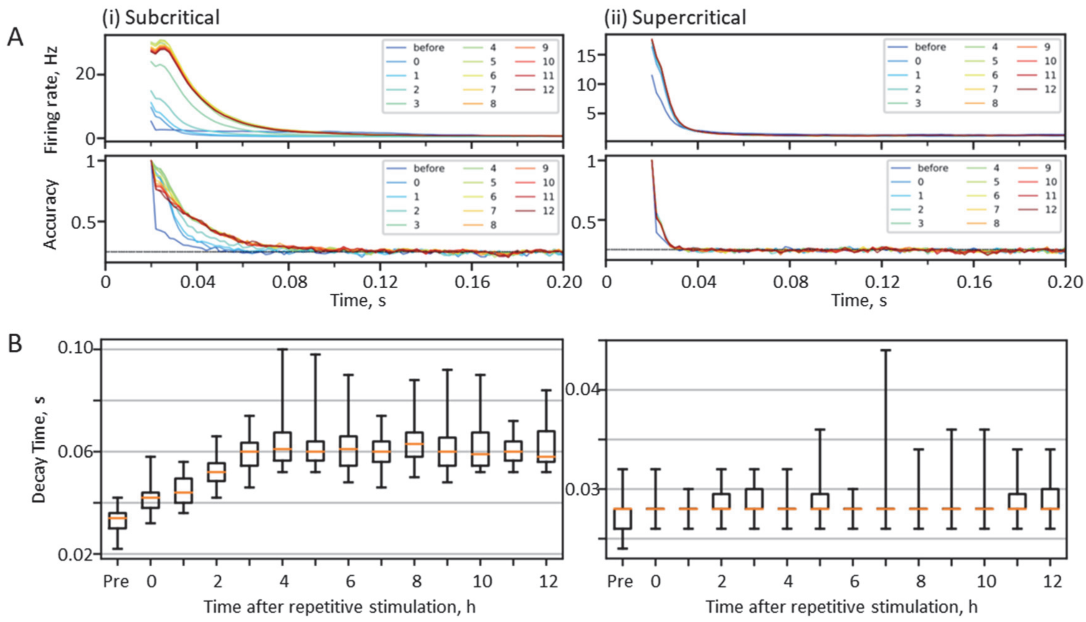

Supplementary Fig. S3 Fading memory in noncritical SNNs. (A) Traces of firing rate and decoding accuracy at the indicated times after repetitive stimulation. (i) Subcritical SNN. (ii) Supercritical SNN. (B) Traces of the fading memory decay time after repetitive stimulation.

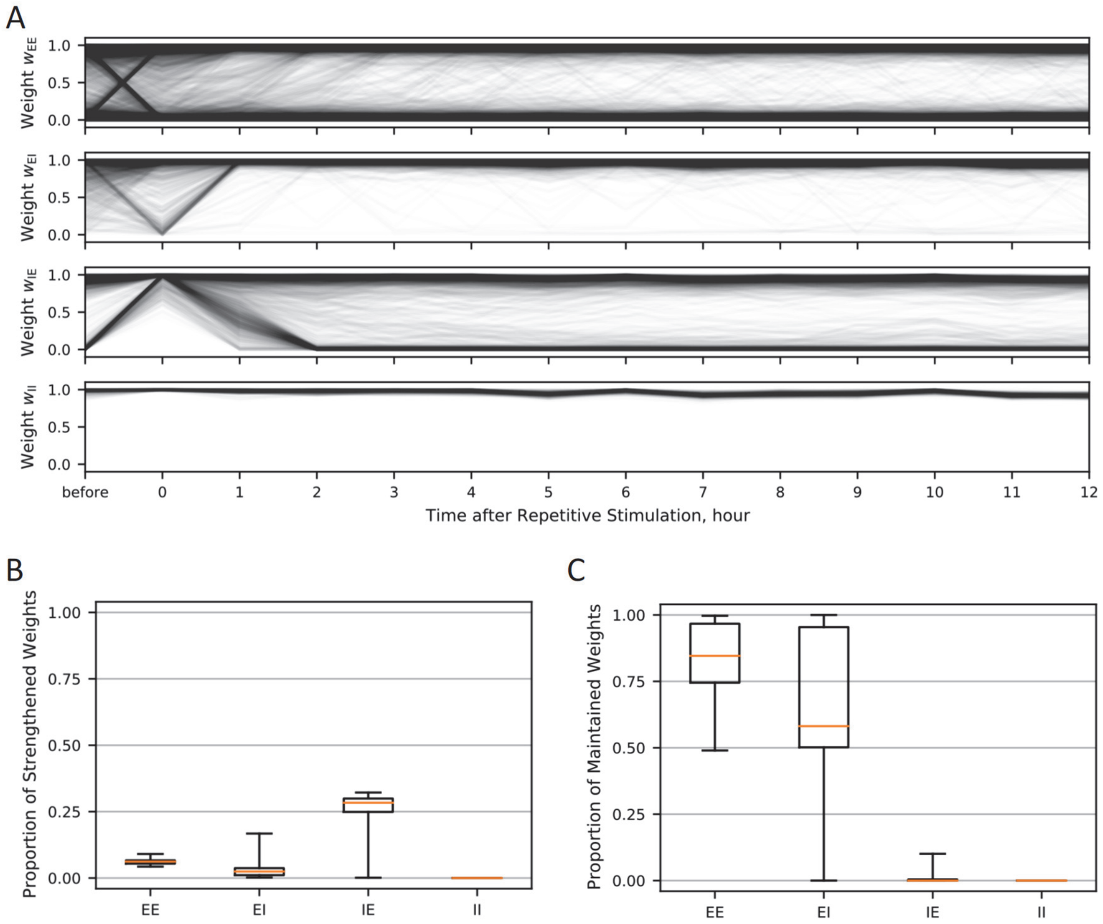

Supplementary Fig. S4 Repetitive stimulation-induced synaptic weight changes in the critical SNN. (A) Weight change over time for w_{EE}, w_{EI}, w_{IE}, and w_{II}. (B) Proportions of weights strengthened by repetitive stimulation. (C) Of the strengthened weights, the proportions of weights were maintained at 0.5 or higher for 12 h after repetitive stimulation.

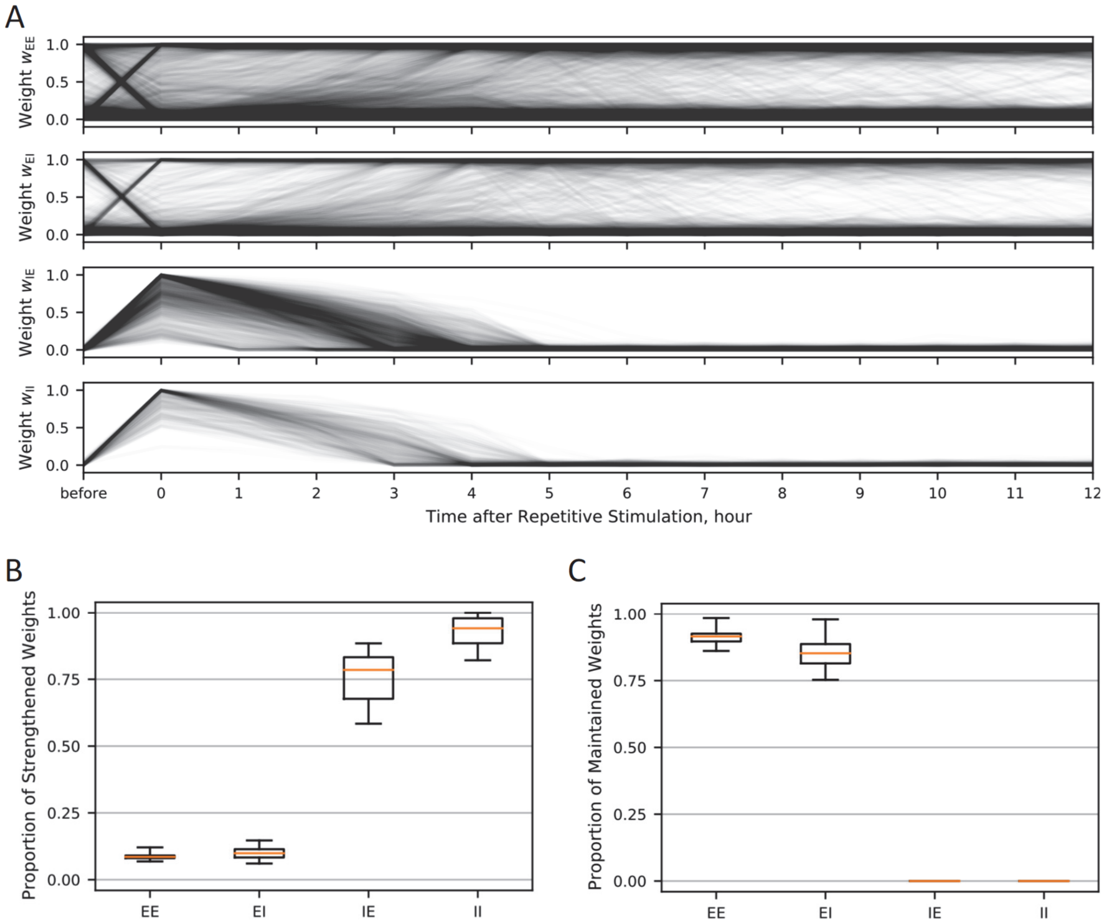

Supplementary Fig. S5 Repetitive stimulation-induced synaptic weight changes in the subcritical SNN. These conventions are in accordance with Supplementary Fig. S4.

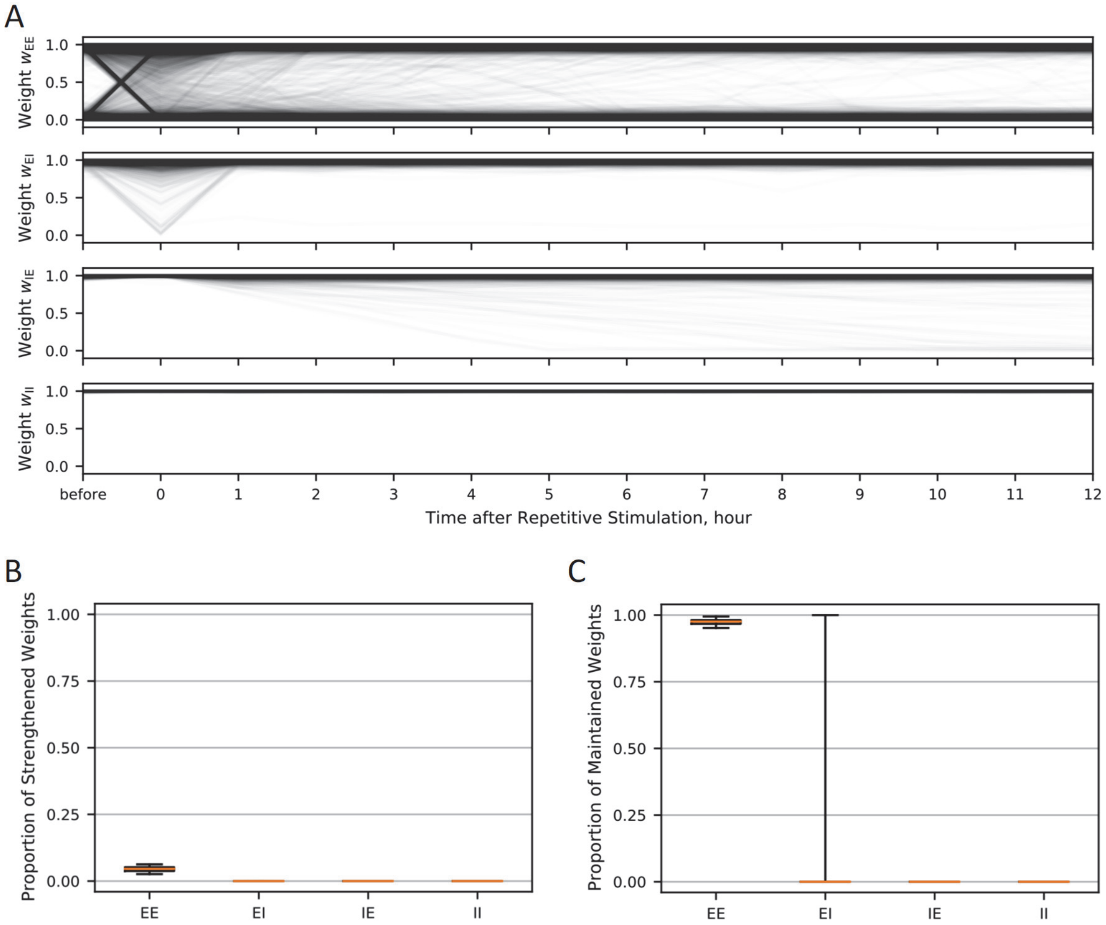

Supplementary Fig. S6 Repetitive stimulation-induced synaptic weight changes in supercritical SNN. These conventions are in accordance with Supplementary Fig. S4.

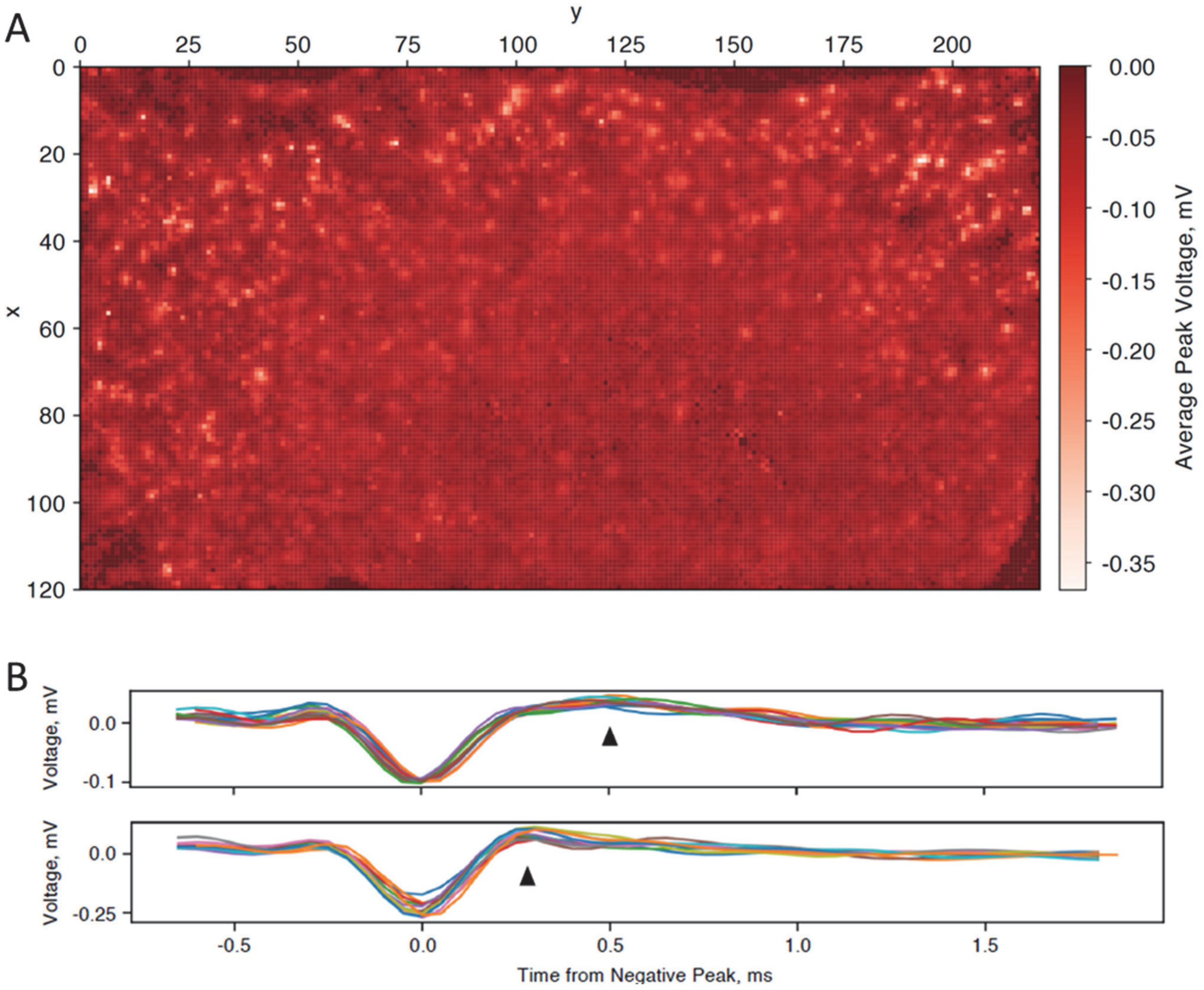

Supplementary Fig. S7 Mapping of action potentials. A. Spatial map of the negative peak of averaged action potential. Each pixel corresponds to an electrode location. B. Representative waveforms of action potentials. Action potentials from a putative excitatory neuron (upper inset) had a more delayed positive peak (arrow) than those from a putative inhibitory neuron (lower inset). Putative excitatory neurons were defined as having a duration of 0.5 ms or more between the negative and positive peaks.

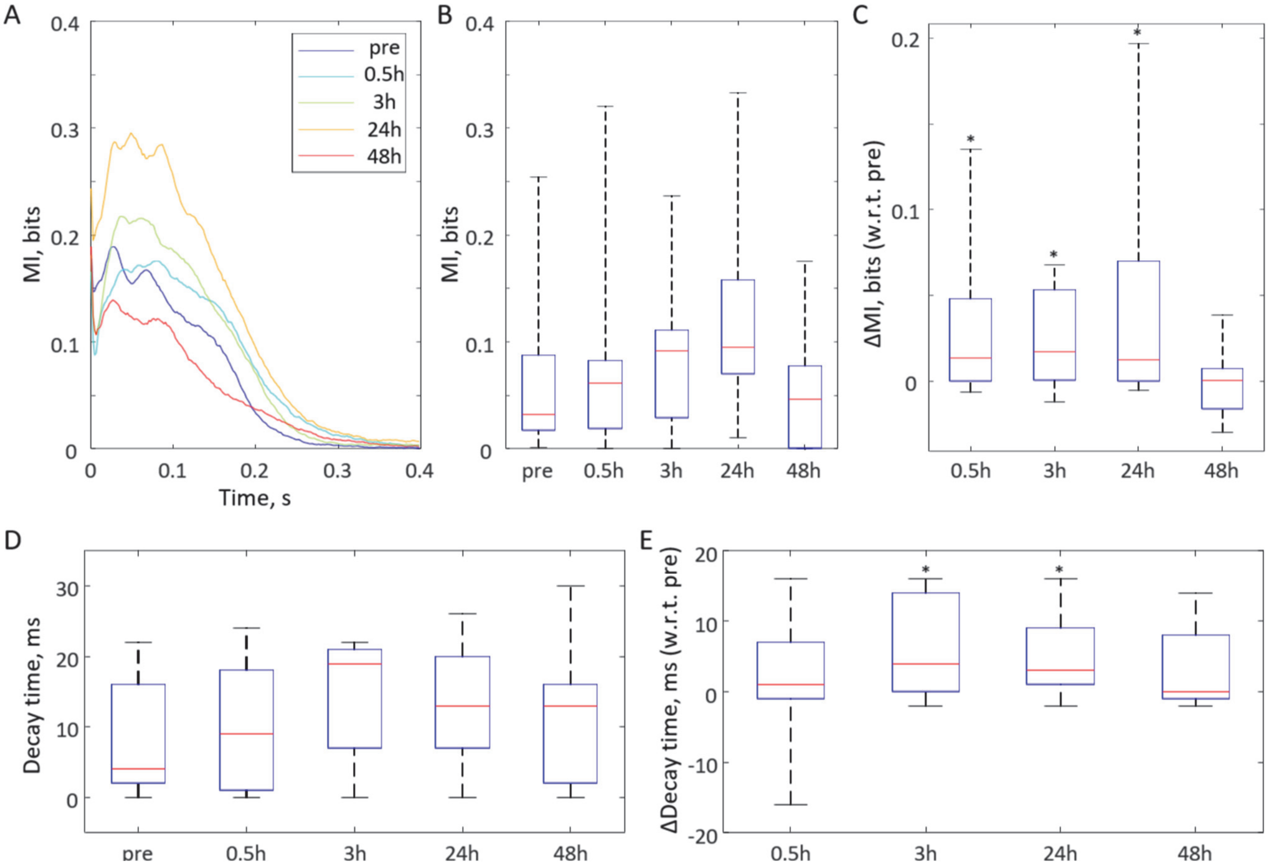

Supplementary Fig. S8 Mutual information (MI) between stimulus patterns and neural activity[1]. (A) Median of MI as a function of time. (B) Boxplot of MI averaged between 0- and 0.4-s post stimulus latency. (C) Increase in MI after spontaneous activity compared to MI in the pre-stimulus session. Asterisks indicate significant increase (Wilcoxon signed-rank sum test: 0.5h, p=0.0231; 3h, p=0.0314; 24h, p=0.0231). (D) Boxplots of the MI decay time. The MI decay time was defined as the post-stimulus latency at which MI falls to the chance level, i.e., no significant difference was observed with respect to MI in a pre-stimulus window (Wilcoxon signed-rank sum test, p>0.05). (E) Increase in MI decay time with respect to that in the pre-stimulus session. Asterisks indicate significant increase (3h, p=0.0228; 24h, p=0.0257)

---

[1] Mutual information $I(S;\, R)$ is given by

$$I(S;R) = \sum_{s\in S, r\in R} p(s,r)\log_2 \frac{p(s,r)}{p(s)p(r)},$$

where S is the set of stimulus patterns, R is the set of firing rates, $p(s)$ and $p(r)$ are the marginal probabilities of stimulus patterns and firing rates, respectively, and $p(s, r)$ is the joint probability of them. $I(S;R)$ was derived at each time bin using the Python library Scikit-learn. $I(S;R)$ was obtained with a moving window of 20-ms bin and 2-ms interval.
